# Strategies for Reliable Exploitation of Evolutionary Concepts in High Throughput Biology

**DOI:** 10.4137/ebo.s597

**Published:** 2008-05-08

**Authors:** Anthony Levasseur, Pierre Pontarotti, Olivier Poch, Julie D. Thompson

**Affiliations:** 1 Phylogenomics Laboratory, EA 3781 Evolution Biologique, Université de Provence, 13331 Marseille, France; 2 Département de Biologie et Génomique Structurales, Institut de Génétique et de Biologie Molculaire et Cellulaire, (CNRS/INSERM/ULP), BP 10142, 67404 Illkirch Cedex, France

**Keywords:** phylogenetic inference, systems biology, evolutionary informatics, information network, functional annotation

## Abstract

The recent availability of the complete genome sequences of a large number of model organisms, together with the immense amount of data being produced by the new high-throughput technologies, means that we can now begin comparative analyses to understand the mechanisms involved in the evolution of the genome and their consequences in the study of biological systems. Phylogenetic approaches provide a unique conceptual framework for performing comparative analyses of all this data, for propagating information between different systems and for predicting or inferring new knowledge. As a result, phylogeny-based inference systems are now playing an increasingly important role in most areas of high throughput genomics, including studies of promoters (phylogenetic footprinting), interactomes (based on the presence and degree of conservation of interacting proteins), and in comparisons of transcriptomes or proteomes (phylogenetic proximity and co-regulation/co-expression). Here we review the recent developments aimed at making automatic, reliable phylogeny-based inference feasible in large-scale projects. We also discuss how evolutionary concepts and phylogeny-based inference strategies are now being exploited in order to understand the evolution and function of biological systems. Such advances will be fundamental for the success of the emerging disciplines of systems biology and synthetic biology, and will have wide-reaching effects in applied fields such as biotechnology, medicine and pharmacology.

## Introduction

The genetic information encoded in the genome sequence contains the blueprint for the potential development and activity of an organism in its environment. This information can only be fully comprehended in the light of the evolutionary events (duplication, gain, loss, recombination, etc.) acting on the genome, that are reflected in changes in the chromosomal organization, the sequence, structure and function of the gene products (nucleic acids and proteins) and ultimately, in the biological complexity of the organism. The recent availability of the complete genome sequences of a large number of model organisms, together with the immense amount of data being produced by the new technological breakthroughs in high-throughput biology, means that we can now begin comparative analyses to understand the mechanisms involved in the evolution of the genome and their consequences in the study of biological systems. At the same time, theoretical advances in information representation and management have revolutionized the way experimental information is collected, stored and exploited. Ontologies, such as Gene Ontology (Ashburner et al. 2000) or Sequence Ontology (Eilbeck et al. 2005), provide a formal representation of the data for automatic, high-throughput data parsing by computers. These ontologies are being exploited in the new information management systems that are being developed to allow large scale data mining, pattern discovery and knowledge inference (e.g. Gouret et al. 2005; Thompson et al. 2006; Gopalan et al. 2006). The new genomic data, combined with recent advances in phylogenetic theory and in informatics, now offers a new global view of the function of living systems across the tree of life (Wolfe and Li, 2003; Doolittle, 2005; Koonin and Wolf, 2006).

It is generally accepted that genome sequences are ideal tools for the study of evolution and for the reconstruction of the tree of life (for a recent review see Delsuc et al. 2005). However, it is perhaps less well accepted that evolutionary analysis represents a powerful tool in the analysis of genomic data. In this review, we will focus on the use of multi-species comparisons and evolutionary approaches for performing comparative data analyses, for propagating information between different systems and for predicting or inferring new knowledge. One of the main advantages of using evolutionary methods in high throughput analyses is that they are designed to represent the causal processes underlying observations. Thus, while some bioinformatics methods distinguish between orthologs and paralogs based on a pattern (e.g. orthologs found by mutual best Blast hits), the evolutionary approach makes this distinction relative to inferred events of speciation, gene duplication and gene loss, based on the reconstruction of a phylogenetic tree. Thus, evolutionary analysis yields inferences, not about patterns, but about the causal factors underlying the patterns. For instance, to identify regulatory elements by “phylogenetic footprinting”, the goal is not merely to identify regions where sequences show high similarity, but to identify regions where selective constraints have resulted in a low rate of evolution. Another area where phylogeny-based inference has been applied is in annotation of protein function in whole genome analyses (Thorne, 2000; Eisen et al. 2002), comparative genomics (Sicheritz-Pontén and Andersson, 2001; Daubin et al. 2002), and in the reconstruction of the evolutionary history of a segment of the human genome (Vienne et al. 2003). It has been shown recently how an explicitly evolutionary approach eliminates certain categories of error that arise from gene duplication and loss, unequal rates of evolution, and inadequate sampling, (e.g. Eisen, 1998; Zmasek and Eddy, 2002). There are now relatively sophisticated analysis tools to address these problems, particularly the problem of identifying paralogy (reviewed in Koonin, 2005). Such methods can be improved by evaluating a more precise model, that has fewer assumptions and that more closely reflects the mechanisms of evolutionary change (Shapiro et al. 2006). Thus, phylogeny-based inference systems are playing an increasingly important role in most areas of high throughput genomics, including studies of promoters (‘phylogenetic footprinting’), interactomes (notion of ‘interologs’ based on the presence and degree of conservation of counterparts of interacting proteins), and also, in comparisons of transcriptomes or proteomes (notion of phylogenetic proximity and co-regulation/co-expression).

Nevertheless, while powerful tools exist for some applications of evolutionary analysis, they remain under-utilized because of the lack of an appropriate informatics infrastructure that makes evolutionary approaches relatively inaccessible and difficult to use. The large-scale organization of sequences into groups related in evolution is not a trivial undertaking and requires the careful selection of methods for aligning sequences and inferring phylogenetic relationships. Considerations include both the applicability of a particular method to the data (e.g. different models of evolution, different degrees of divergence) and the practical consideration of computational feasibility. Here we will review the recent developments in the field, aimed at making automatic reliable phylogeny-based inference feasible in large-scale projects. We will then discuss how evolutionary concepts and phylogeny-based inference strategies are now being exploited in high throughput biology projects in order to understand the evolution and function of biological systems.

## Methods for Automatic, Reliable Phylogeny-Based Inference

**Figure 1:**
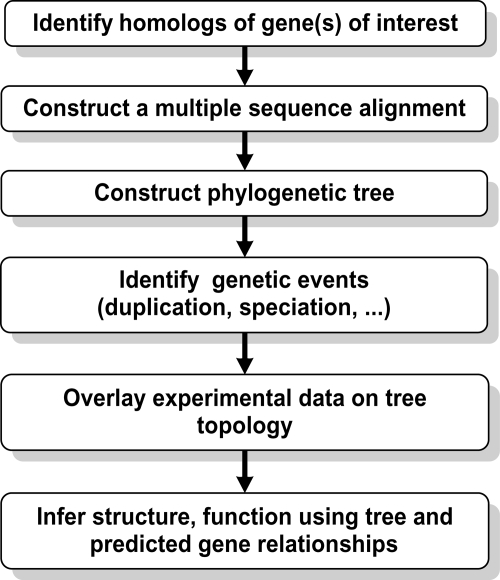
General principles of a phylogenetic inference strategy.

Construction and exploitation of phylogenetic trees and understanding of evolutionary events are very complicated tasks, but recent developments constitute major advances that address many of the major bottlenecks. The general strategy, outlined by Eisen in 1998, is shown in Figure 1. First, an evolutionary analysis depends on a presumption of homology. In molecular sequence analysis, this corresponds to the dual task of finding homologs by performing similarity searches in sequence databases, and of identifying homologous residues in a multiple sequence alignment. Next, a phylogenetic tree is constructed and the tree topology is analyzed to localize speciation or gene duplication events at particular branch points. Finally, the phylogenetic tree is overlaid with experimental data, and changes in structure or function can be traced along the evolutionary tree.

Such an evolutionary approach provides a general framework that can be applied effectively to many different kinds of data, including complete genome sequences, cDNAs or ESTs, RNA or protein sequences, or even whole-genome features beyond the sequence level, such as gene order (synteny) or gene content (i.e. the specific genes found in a genome). However, generally speaking, protein sequences have been shown to be better than nucleotide sequences in obtaining the true tree topology or trees close to the true tree (Russo et al. 1996).

### Selection of homologous sequences

The first step in any phylogenetic analysis generally requires the identification of sequences related to the genes of interest. The goal is to include sufficient diversity for optimal information content, since distantly related sequences can help many aspects of the analysis. Nevertheless, the sequences should share sufficient residue identity to enable the generation of an accurate multiple sequence alignment and phylogenetic tree, otherwise noise is introduced in the analysis. For protein sequences, it is generally considered that two sequences sharing over 30% identity will share a common fold and similar function, but more sensitive methods have now been developed to detect potential evolutionary relationships in the twilight zone, below 30% identity e.g. Gen-Threader (ideal for automatically predicting the structure of all the proteins in a translated bacterial genome) (McGuffin and Jones, 2003), SAM-T99 (begins with a single target sequence and iteratively builds a hidden Markov model from the sequence and homologs (Karplus et al. 1998) and PSIBLAST (Altschul et al. 1997). Given a seed sequence, PSIBLAST iteratively searches a sequence database to identify and align putative homologs from which a profile (PSSM) is constructed for database search in the next iteration. PSIBLAST thus provides essential information about local sequence similarities, which might lead to evolutionary clues about the structure and/or function of the query sequence. Other programs have been developed recently for more specific tasks, such as very rapid large-scale mRNA/DNA alignments e.g. BLAT (Kent, 2002) or the identification of novel noncoding RNAs in genome sequences (e.g. Washietl et al. 2005).

### Construction of high quality, reliable multiple alignments

Once the set of potential homologs has been identified, the next step is to construct a multiple sequence alignment. A vast array of diverse algorithms has been developed in an attempt to construct reliable, high-quality multiple alignments within a reasonable time limit that will allow high-throughput processing of large sequence sets. Traditionally the most popular method has been the progressive alignment procedure (Feng and Doolittle, 1987), which exploits the fact that homologous sequences are evolutionarily related. A multiple sequence alignment is built up gradually using a series of pairwise alignments, following the branching order in a phylogenetic tree. A number of different alignment programs based on this method exist, based either on a global alignment algorithm that aligns the sequences over their full lengths, notably ClustalW/X (Chenna et al. 2003), or on a local alignment algorithm, that focuses on shared regions of high similarity and ignores regions that do not show clear sequence homology. A comparison of a number of local and global protein alignment methods based on the BAliBASE benchmark (Thompson et al. 1999) showed that no single algorithm was capable of constructing accurate alignments for all test cases. A similar observation was made in another study of RNA alignment programs (Gardner et al. 2005), where algorithms incorporating structural information outperformed pure sequence-based methods for divergent sequences. Therefore, recent developments in multiple alignment methods have tended towards integrated systems bringing together knowledge-based or text-mining systems and prediction methods with their inherent unreliability. Some of the most widely used or more innovative methods include: DbClustal (Thompson et al. 2000) that was developed to align sets of sequences detected by a BlastP homology search, TCoffee (Notredame et al. 2000), MAFFT (Katoh et al. 2002), MUSCLE (Edgar, 2004) and Probcons (Do et al. 2005). These programs combine the advantages of both local and global alignment algorithms and generally incorporate an iterative refinement strategy. In comparisons based on the latest version of BAliBASE (Thompson et al. 2005a), the best alignments in all the alignment tests were achieved by TCOFFEE and PROBCONS, although a large time penalty was incurred. The programs MAFFT and MUSCLE obtained the next highest scores, with a significant reduction in the time required to produce the alignments. Nevertheless, for all the programs tested, a decrease in accuracy of the alignments with decreasing residue identity is clearly demonstrated, with a significant loss occurring for highly divergent sequences (<20% identity), which corresponds to the ‘twilight zone’ of evolutionary relatedness.

Although much progress has been achieved, the latest methods are not perfect and misalignments can still occur. If these misalignments are not detected, they will lead to further errors in the subsequent applications that are based on the multiple alignment. The assessment of the quality and significance of a multiple alignment has therefore become a critical task, particularly in high-throughput data processing systems, where a manual verification of the results is no longer possible. Multiple alignment validation is difficult because the true alignment of naturally evolved sequences is never known. As an alternative solution, a number of quality assessment (QA) measures have been proposed, known as objective functions, that estimate how close the alignment is to the correct or optimal solution. Until recently, the most widely used alignment quality measures were based on the sum-of-pairs score (Carrillo and Lipman, 1988) or a log-likelihood ratio, such as relative entropy (Hertz and Stormo, 1999). Other scores e.g. NorMD (Thompson et al. 2001) or MUMSA (Lassmann and Sonnhammer, 2005) can be used to assess the significance of a given multiple alignment and provide a practical quality filter in large scale automatic or semi-automatic genome annotation pipelines. All these objective functions calculate a global score that estimates the overall quality of a multiple alignment. However, even when misalignments occur, it is not necessarily true that all of the alignment is incorrect. Useful information could still be extracted if the reliable regions in the alignment could be distinguished from the unreliable regions. The prediction of the reliability of specific alignment positions has therefore been an area of much interest, e.g. AMAS program (Livingstone and Barton, 1993), Al2Co (Pei and Grishin, 2001), DIVAA (Rodi et al. 2004), and for nucleic acid sequences, the ConFind program (Smagala et al. 2005). Regions that are doubtful should be excluded from the subsequent phylogenetic analysis. Alignment columns for which a substantial number of sequences (e.g. >20%) contain only gap characters are also worthwhile removing.

### Construction of phylogenetic trees

A phylogenetic tree shows the evolutionary relationships among different species or other entities that are believed to have a common ancestor. The output tree of a phylogenetic analysis based on sequenced genes is an estimate of the gene’s phylogeny (i.e. a gene tree) and not the phylogeny of the taxa (i.e. species tree) from which these characters were sampled (Page and Michael, 1997). Sometimes a gene tree disagrees with the species tree (constructed for example from anatomical and paleontological considerations) due to gene duplication, loss, and lineage sorting. Therefore, species phylogenies are now more commonly obtained by applying consensus tree/supertree methods to collections of gene trees (Sanderson and Driskell, 2003).

In this section, we will concentrate mainly on the reconstruction of gene trees, since these are more generally used in structural/functional inference approaches. At this point, an important point has to be underlined: a protein is often composed of different domains and these domains may have different evolutionary histories due to genomic recombinations and exon shuffling (Schmidt and Davies, 2007). Such events cannot be identified based on the alignment alone and a phylogenetic analysis at the individual domain level is essential, since the topologies of the phylogenetic trees corresponding to the two domains may be different. In the case where the resulting domain phylogenies are in fact congruent, the phylogenetic signal can be combined into a single gene phylogeny.

Once the domain structure of the gene has been identified, there are two main classes of phylogenetic tree construction methods: distance based (neighbor joining) and character based (maximum parsimony, maximum likelihood and Bayesian method) (reviewed in Brocchieri, 2001). Distance-based methods compute a matrix of pairwise distances between sequences in an alignment and thereafter ignore the sequences themselves, constructing a tree based entirely on the original distance computation. The computation of the character-based distance can be calculated using different matrices. These matrices use maximum likelihood estimates based on family alignments (e.g. Dayhoff PAM matrix model, JTT matrix model), or a model based on the genetic code together with a constraint on changing to a different category of amino acid. The distances can also be corrected for gamma-distributed and gamma-plus-invariant-sites-distributed rates of change in different sites. Rates of evolution can vary among sites in a pre-specified way, and also according to a Hidden Markov model.

Unfortunately no biological datasets exist to assess phylogenetic tree methods directly. The community has therefore no way of knowing the true evolutionary tree underlying a protein superfamily. For this reason all experimental validations of phylogenetic inference methods have been performed on simulated data and results relevant to protein superfamilies are inconclusive (Sjolander, 2004). One approach to tackle this problem, is to combine different methods [e.g. Figenix (Gouret et al. 2005) combines neighbour joining, maximum parsimony and maximum likelihood] to calculate the trees. Given the same multiple sequence alignment, two reconstruction methods will produce at least two trees and sometimes many more (for example the maximum parsimony tree will produce many hundreds of equally parsimonious trees). Closely related subgroups are found reliably by most tree methods and most of the differences between trees are found at the deeper nodes in the tree. To avoid any systematic biases of one particular method, bootstrap analysis is combined with different tree methods (Brocchieri, 2001). The next step in the Figenix system is to compare the topologies obtained from the different tree methods using a suitable algorithm such as the Hasegawa test (Kishino and Hasegawa, 1989) and to look for congruence of the trees. When the three trees are congruent a fusion is performed, and in the case where one of the trees is not congruent with the others, only two trees are fused. In the case where the three trees are not congruent, no fusion is possible and the default choice is then the maximum likelihood tree.

The phylogenetic reconstruction process described above also allows the possibility of inferring the sequences of ancient ancestors of modern species using a model of molecular evolution (reviewed in Danchin et al. 2007). This ancestral sequence reconstruction works for the evolution resulting from a substitution process and can be performed at the protein or at the DNA gene sequence level. Reconstruction can also be made from large genomic regions, for example Blanchette et al. 2004 proposed *in silico* reconstruction of a 1.1 Mb around the *CFTR* locus of the eutherian ancestral genome. Computational simulations were performed demonstrating that large parts of the euchromatic genome from early eutherian could be accurately reconstructed when specific extant mammalian genomes were carefully chosen. Using ~20 modern mammals, the authors expected to achieve 98% correct bases in reconstructing megabase-scale euchromatic regions of the eutherian ancestral genome. Mutational processes such as tandem and segmental duplication, inversion, and translocation or different modes of selection were not included in the simulation, as no models were available, in contrast to amino acid or nucleotide substitution. However reconstructions have been made for the other genetic events using less realistic evolutionary models.

### Ortholog/paralog information

The next step is to differentiate between true orthologs (homologous genes resulting from speciation) and paralogs (homologous genes resulting from duplication) among sequences in the tree. Several approaches not based on phylogenetic analysis claim to find orthology. One of the most popular is based on a clustering method such as Inparanoid (Remm et al. 2001). The clustering requires a complete genome and gives erroneous information in the case of lineage-specific differential paralog loss (see for example Danchin et al. 2006). This is not the case for ortholog and paralog identification based on phylogeny. When phylogenetic trees are constructed, specific algorithms are applied to distinguish between orthologs and paralogs, (e.g. Zmasek and Eddy, 2002; Dufayard et al. 2005).

In general, orthologs are considered to have more chance of sharing a similar function compared to paralogs (e.g. Collette et al. 2003). This can also be argued theoretically since after duplication, either one of the copies is lost, or both duplicates undergo sub-functionalization, or one of the duplicates evolves toward a new function (neo-functionalization) (Force et al. 1999). By function, Force et al. meant either biochemical function or expression pattern meaning that a functional shift corresponds, for the authors, either to a functional biochemical shift or a transcriptional shift. At the molecular level, paralogs can be either biochemically sub-functionalized or neo-functionalized and they will have therefore a different biochemical function, although in the case of neo-functionalization one of the copies will retain the ancestral function. Note that the paralog that undergoes neo-functionalization can be identified by the evolutionary shift analysis (see below). At the transcriptional level, in the case of neo-transcription events, one of the copies will retain the ancestral transcription pattern. In the case of sub-transcription, the two copies will have a complementary pattern that will recapitulate the patterns of the preduplicate copy and the non duplicate ortholog.

### Analysis of patterns of conservation/ divergence, detection of genomic content submitted to positive selection

Analyses of evolutionary change at the amino acid and nucleotide level provide valuable hints of what is happening at the molecular level in biological systems. Patterns of replacement, observed in sequence alignments, can reflect residues important for function, stability, and folding (reviewed in Clifford et al. 2004). For example, the functional importance of sites is intuitively inversely related to the evolutionary rate of amino acid replacements. This intuition arises from one interpretation of the neutral theory of evolution in which the site of the greatest functional significance are under the strongest selective constraint (Gu, 2003). An organism that experiences a replacement at one of these sites is less likely to survive and therefore to reproduce. In some cases the extent to which function constrains the evolution of a protein sequence can be estimated by measuring the ratio of non-synonymous (replacement) to synonymous (silent) substitutions during evolution (Liberles and Wayne, 2002). This ratio is also used to detect positive selection in coding DNA which in turn could be linked to a functional shift. To assess more broadly the possible functional significance of sequence evolution, particularly among distantly related proteins, other approaches have emerged that consider amino acid replacements (non-synonymous substitution) alone (Gaucher et al. 2002). Finally, analysis of the population genomic variation provides an alternative scheme that allows the detection of genomic content submitted to positive selection (Biswas and Akey, 2006). These approaches are reviewed in more detail in the following sections.

#### Methods based on amino acid replacement

These methods begin by analyzing how the evolutionary rates of amino acid replacements differ among sites in a protein sequence (site to site rate heterogeneity), with a statistical formalism in which the rate varies among sites according to a gamma distribution (Yang, 1996). In a conventional analysis of sequence evolution using the gamma model, termed homogeneous, rapidly and slowly evolving sites remain rapid or slow across the entire evolutionary tree. Such a homogeneous evolutionary rate is expected when the functional constraints at sites are constant for the entire evolutionary history. However if the function of the protein is changing, some residues might be subjected to altered functional constraints in various places of the phylogenetic tree, which implies that the evolutionary rates at these sites will be different in different branches of the tree (heterotachy). To model this phenomenon, a non-homogeneous gamma model is used, where the constraint of fixed rates per site along the phylogeny is relaxed to allow the identities of fast and slow sites to change over time i.e. to allow site specific rate shifts (Gu, 2003). Rate shifted sites then correspond to the residues that have either enhanced or reduced selective constraint as a possible consequence of the change of function during protein evolution (Lopez et al. 2002).

#### Comparison of silent and replacement sites

Another possible effective approach is to compare the rates at which synonymous (silent) d_S_ and non synonymous (replacement) d_N_ mutation are fixed in the history of a given gene. The silent rate d_s_, provides a benchmark against which we can decide whether the replacement rate d_N_ is accelerated or diminished possibly by natural selection on the protein (Miyata and Yasunaga, 1980). Thus d_N_ <d_S_, d_N_ = d_S_, dN>dS, represent negative (purifying) selection, neutral evolution and positive selection respectively. A problem with this criterion is its lack of discriminative power (Yang, 2005). Most proteins have highly conserved regions where replacements are not tolerated and d_N_ is almost 0. Thus, comparison of a pair of genes, by averaging the d_N_ and d_S_ rates over all sites in the protein, fails to infer positive selection, because the signal of positive selection is overwhelmed by the ubiquitous purifying selection. To boost the power of the detection method, more recent work has focused on detecting selection that affects individual sites rather than the whole protein, or particular lineages rather than the whole phylogeny. Nevertheless, it is important to note that synonymous substitutions are generally neutral and therefore occur at a relatively rapid rate. Hence the d_N_ over d_S_ ratio can only be used to detect recent functional divergence, as synonymous sites rapidly become saturated with mutations. For a typical vertebrate nuclear encoded gene, this type of analysis has been generally useful only as far back as around 150 million years ago (Gaucher et al. 2002). Nevertheless it should be noted that these methods have been used in a few cases to detect older events (Rodriguez-Trelles et al. 2003; Bos, 2005).

The positively selected sites identified by the methods described above can be further evaluated for their roles in functional divergence by mapping them onto the available tertiary (or three-dimensional) structures of the protein (Blouin et al. 2003). Mutagenesis experiments can also be performed to unambiguously demonstrate that the positively selected sites are indeed involved in the functional shift, which is a *sine qua non* condition to clearly establishing a connection between such evolutionary and functional shifts (Levasseur et al. 2006). It should be noted however that few examples of relaxed or positive selection have been linked to actual functional shifts due to a specific environmental change. (Levasseur et al. 2007).

#### Signatures of positive selection in populations

At the population scale, targets of positive selection can be used to shed light on the historical forces that have shaped the genomic content of a population. In contrast to the neutral model of evolution, positive selection might affect the genetic variation in the allele frequency distribution or perturb the degree of linkage disequilibrium. The identification of a signature of positive selection is challenging when only one locus is studied, because of the confusing effects of population demographic history versus natural selection. Therefore numerous loci spanning the genome are taken into account to detect unusual patterns of genetic variation. A great deal of effort has been devoted to the development of methods to detect positive selection in populations (reviewed in Biswas and Akey, 2006). Among these methods, two different tests can be mentioned: those based on polymorphisms within species and those based on polymorphisms within species combined with the divergence between species. The polymorphism-based methods involve sampling of multiple copies of orthologous genomic regions within populations to detect single and recent selective sweeps. Divergence-based methods involve sampling single individuals from each species and then testing for site changes that occurred more often than expected across the species tree. The use of these approaches should lead to a better understanding of the ecological context in which a species is constrained and has evolved, that in turn could be informative for the study of adaptation at the molecular level (Ronald and Akey, 2005). Despite the few examples reported in the literature, such positively selected genes could be indirectly linked to particularly important functions related to environmental changes.

### Identification of the evolutionary histories of other genetic events

The principal genetic events that determine genome shape and structure are believed to be gene duplication, gene loss, horizontal gene transfer (HGT), and chromosomal rearrangements, such as inversions, translocations and duplications, that range from part of a gene to hundreds of genes. Assuming a particular species tree topology, methods of evolutionary analysis can be used to map these different types of genetic events onto the branches of the tree. For example, phylogenetic trees were systematically analyzed for the presence of gene duplication events at different points during vertebrate evolution (Blomme et al. 2006). Duplication events were evaluated by relative dating, based on the relative position of the duplicated genes compared to speciation events in the phylogenetic tree. Gene loss following gene duplication events was then estimated as parsimoniously as possible. In another study (Fong et al. 2007) protein domain architectures for a wide range of organisms were mapped to the NCBI taxonomy, in order to identify the evolutionary pathways by which extant architectures may have evolved. They proposed a model of evolution in which domain architectures arose through rearrangements of inferred precursor architectures and acquisition of new domains.

With the sequencing of numerous complete genomes, it is now possible to reconstruct phylogenies based on whole genome data (reviewed in Wolf et al. 2002). Whole genome comparisons are also being used to study large-scale mechanisms, such as chromosomal rearrangements, to detect syntenic regions, i.e. blocks of genes or other markers with evolutionary conserved order, and to reconstruct ancestral genomes. Several *in silico* approaches have been used to find conserved regions also called conserved homologous synteny blocks (HSB), for example, the initial GRIMM-synteny algorithm identifies HSB from sequence alignment or from localized orthologs (Pezner and Tesler, 2003). Another example is the universal E-painting tool (electronic chromosome painting) (Kohn et al. 2006). In this method, the genes and their chromosomal assignment for each species are considered and HSB can be assigned according to a user-specified species. Ancestral reconstructions can then be inferred from the genomes of modern species using a model of molecular evolution. Four methods are commonly used and are briefly described below.

- The cladistic method is based on comparative analysis of ancestral *versus* derived features using appropriate outgroup species (Dobigny et al. 2004; Henning, 1966). Thus, a feature is considered ancestral if the trait is found not only within a given taxon but also in more distantly related species that serve as out-groups. The identification of ancestral features is aided by parsimonious analyses of potential evolutionary rearrangement events.
- GRIMM (Genome Rearrangements In Man and Mouse; note that despite the name this method can be used for reconstruction of all species), is also based on a parsimonious method. GRIMM implements the Hannenhalli-Pevzner algorithm to compute the reversal distance between two unichromosomal genomes, and Tesler’s algorithm for computing the distance between two multichromosomal genomes (Tesler, 2002a, b). The reversal distance between two genomes is the minimum number of reversals it takes to transform one genome to another. For unichromosomal genomes, the rearrangement events considered are reversals (also known as inversions), whereas for multi-chromosomal genomes they can be reversals, translocations, fissions, and fusions.
- MGR (Multiple Genome Rearrangement) implements an algorithm that, given a set of genomes (at least three), seeks a tree such that the sum of the rearrangements is minimized over all the edges of the tree (Bourque and Pevzner, 2004). It can be used for phylogeny inference and also for inference of ancestral gene orders.
- CARs (contiguous ancestral regions) aims to infer segment order in the ancestral genome by formalizing the problem using graph theory from a provided phylogenetic tree where each leaf corresponds to a genome (Ma et al. 2006). The algorithm identifies a most-parsimonious scenario for the history of each individual adjacency, and weights are attributed to the graph edges to model the reliability of each adjacency. A heuristic algorithm finds sets of paths (corresponding to contiguous ancestral regions) in the graph covering maximum total weights.

### Integrating evolutionary information in the genomic information network

As more whole-genome projects are being completed, postgenomic biology is providing insight into the function of biological systems by the use of new high-throughput bioanalytical methods, information technology, and computational modelling; an emerging discipline known as systems biology. Traditionally, the information produced by bioinformatics studies was interpreted by a human expert who had the experience necessary to understand the patterns revealed by the computational analyses. In the post-genomic era, the volume of data available requires automatic processing by ‘intelligent’ computer systems that are capable of understanding the relations and patterns hidden in the data. Inferring new knowledge by combining different kinds of “post-genomics” data obviously necessitates the development of new approaches that allow the integration of variable data sources into a flexible framework. The first step to achieve this is to represent the basic knowledge in the domain of interest in a format that can be understood by the computer. Ontologies provide an ideal means of representing the fundamental concepts in a domain and the relationships that exist between them. They are used for communication between people and organisations by providing a common terminology over a domain. But perhaps the most important aspect of an ontology is that provide the basis for interoperability between different databases and computational systems.

The most well known biological ontology is the Gene Ontology (GO) (Ashburner et al. 2000), which has become the *de facto* standard for describing the principal attributes (the molecular function, biological process, and cellular component) of knowledge about gene products. GO is part of an umbrella project, called Open Biomedical Ontologies (http://obo.sourceforge.net/), whose goal is to provide a set of compatible ontologies, which can be used in combination in order to integrate individual data resources into a coherent whole. The ontologies grouped together at the OBO web site cover a wide range of biomedical fields, such as specific organism anatomies, phenotype characters (PATO), taxonomic classifications or transcriptomic and proteomic experimental protocols and data. Various ontologies have also been developed for particular aspects of molecular sequences, such as gene structure (SO), protein function (GO) or protein—protein interactions (MI). A multiple alignment ontology (MAO) (Thompson et al. 2005b) has also been developed covering both nucleic acid and protein sequence alignments.

These ontologies provide the basis for integration of information resources and as a query model for information management systems that include automated inference and reasoning. The goal of ontology-based information management systems (IMS) is to combine information from different data resources into a unified system, such that the cumulative information provides greater biological insight than is possible if the individual information sources are considered separately. IMS are designed to help biologists systematically gather and exploit all the data crucial for their research, by automating many aspects, from data acquisition to knowledge discovery. For example, GIMS (Genome Information Management System) (Cornell et al. 2003) is an object database that integrates genomic data for *Saccharomyces cerevisiae* with data on the transcriptome, protein-protein interactions, metabolic pathways and annotations, such as gene ontology terms and identifiers. Another example is the MACSIMS information management system (Thompson et al. 2006), for the integration of different types of data in the framework of a multiple sequence alignment. MACSIMS combines knowledge-based methods with complementary *ab initio* sequence-based predictions for protein family analysis. A data collection system automatically retrieves a range of information, from taxonomic data and functional descriptions to individual sequence features, such as structural domains and active site residues. A number of algorithms are included for reliable data cross-validation, consensus predictions and rational propagation of information from the known to the unknown sequences. Thus, structural and functional data can be combined with information about the conservation of the family and the variability observed at different residue sites.

## Exploitation of Evolutionary Concepts and Phylogenetic-Based Inference

The last decade has given us access to the complete genomes of a large variety of organisms. With the completion of the sequencing of the human genome and other model organisms, one of the most important problems to come will be to understand how complex networks function to perform the essential processes of life. At the same time, enormous quantities of biological data are now being produced and collected in large-scale databases generally available via the Internet. Nevertheless, this accumulation of large-scale data is only an indispensable preliminary to the understanding of the principles and fundamental mechanisms of life. A critical stage in this understanding will be the comparative analysis of diverse sequences and the understanding of the evolutionary processes (duplication, loss, recombination) involved, since they determine the sequence, the structure and the function of macromolecules and define, at the highest level, the biological complexity of organisms. Indeed, the evolutionary message currently represents a crucial element for the understanding of complex systems, via the integration and the extraction of knowledge, combined with mathematical modelling and simulation to predict the behaviour of a system under different conditions (Kanehisa and Bork, 2003). Thus, the contributions of the phylogenetic dimension have been particularly important in structural/functional annotations of genes, in the studies of promoters, interactomes, and also in comparisons of transcriptomes or proteomes.

### Structural/functional annotations of genes

At the time of writing, over 1000 genomes (from bacteria, archaea and eukaryota, as well as many viruses and organelles) are either complete or being determined, but biological interpretation, i.e. annotation, is not keeping pace with this avalanche of raw sequence data. There is still a real need for accurate and fast tools to analyze these sequences and, especially, to find genes and determine their functions. The annotation of protein-coding sequences can be split into two complementary tasks, structural annotation and functional annotation.

### Structural annotation

Finding genes in a genomic sequence is far from being a trivial problem. It has been estimated that 44% of the protein sequences predicted from eukaryotic genomes and 31% of the HTC (High-throughput cDNA) sequences contain suspicious regions (Bianchetti et al. 2005). The structural annotation consists in localizing genome features such as protein-coding sequences and then in predicting the intron/exon organization and inferring the sequence of the corresponding protein. This step is very important for the functional annotation, because a missed exon, for example, could be dramatic for the functional inference. The most efficient programs for protein sequence prediction combine *ab initio* along with similarity-based programs (Mathe et al. 2002). However, such programs require that homologous proteins are found in biological databases. When proteins sharing significant similarities are found, this indicates that the proteins could be homologous, which means that they originate from a common ancestral gene. This common ancestor evolved toward the genes coding for these proteins, as well as the other members of the family, by substitution in the coding or the noncoding region, 5′ and 3′ exon extension, by shifts in the acceptor and donor sites, or by exon losses and gains. All these events need to be modeled by the algorithm used for the structural annotation. For prokaryotic genomes, these combined methods are highly successful, identifying over 95% of the genes (e.g. Aggarwal and Ramaswamy, 2002), although the exact determination of the start site location remains more problematic because of the absence of relatively strong sequence patterns. The process of predicting genes in higher eukaryotic genomes is complicated by several factors, including complex gene organization, the presence of large numbers of introns and repetitive elements, and the sheer size of the genomic sequence (for a review, see Zhang, 2002).

### Functional annotation

Ancestrally, a gene product has a given function. This function can change in the daughter genes (gene originating via descent transmission or duplication) due to mutational events on the gene. Following speciation, there are many possible molecular events that can drive the functional divergence, including changes in the coding sequence that lead to shifts in protein function and changes in regulatory regions that affect gene expression or mRNA splicing. These shifts, either in molecular function, sub-cellular localization or transcriptional tissue-specific activity, can be revealed at the biochemical level as well as at the higher levels of organism organization (e.g. cellular processes, physiology or social organization).

As a number of studies have shown, standard methods of gene function prediction have lead to a number of systematic errors (e.g. Devos and Valencia, 2001; Gilks et al. 2002). In most genome annotation projects, the standard strategy to determine the function of a novel gene is to search the sequence databases for homologs and to propagate the structural/functional annotation from the known to the unknown gene. However, most automatic genome projects only use information from the top best hits in the database search, as sequence hits with higher Expect values are considered unreliable. This has lead to a certain number of errors in genome annotations. Gene duplication is perhaps the single greatest contributing factor to errors in function prediction by homology. When gene duplication occurs, one copy must supply the original function, while the other is allowed to evolve novel functions. Paralogous genes, related by duplication events, are more likely to have divergent function, while orthologous genes, related by speciation, are more likely to share a common function. Domain shuffling (Galperin and Koonin, 1998) also complicates matters, as standard methods of homology detection typically ignore whether two proteins align globally or only locally. This can lead to errors in function prediction, as the presence or absence of a domain can have a dramatic impact on protein molecular function. Changes in function due to speciation are a third contributing factor to errors in function prediction. Genes can share a common ancestor, and be orthologous, but still have different functional specificities, particularly if the genes are contained in very distantly related species (Gerlt and Babbitt, 2001).

Phylogenomic analysis, combining phylogenetic tree construction, integration of experimental data and differentiation of orthologs and paralogs, has been proposed to address these errors and improve the accuracy of gene functional annotations. Using sequence shift information, the gene genealogy can be reconstructed and the function genealogy can then be superimposed on the gene genealogy. As more information and more refined methods are available for biological sequence data, reconstructing a tree that deciphers the evolutionary history of genes has become more straightforward and accurate. As a consequence, a number of software platforms have been developed recently, e.g. Figenix (Gouret et al. 2005), SIFTER (Engelhardt et al. 2005), that identify important events in the evolutionary history of a gene, based on a phylogenetic analysis and infer gene function by superimposing experimental information on the phylogenetic tree.

### Identification of non coding functional elements

Beside gene coding sequences, others sequences in the genome are of significant relevance because of their important functions, among them the RNA coding gene, regulatory sequences (promoters, enhancers, silencers …), intron splicing sites and microRNA. A powerful method for discovering non-coding functional elements consists in aligning orthologous genomic sequence from different species, coupled with finding regions of conservation. As for coding sequences, the basic principle behind the method (called phylogenetic footprinting) is found in classical molecular evolution theory. Indeed mutations in functional sites are likely to be deleterious and therefore will be selected against resulting in a reduced rate of evolution in functional elements (for review, see Jones, 2006).

Classical phylogenetic footprinting methods can be separated into two groups. The first group (e.g. Shah et al. 2004; Siepel et al. 2005) is based on the multiple alignment of the orthologous regulatory regions from several species and the subsequent identification of conserved regions in the alignment. The multiple alignments can be based on either local or global methods. Local alignments, e.g. Patternhunter (Ma et al. 2002), can be used for the comparison of whole vertebrate genome assemblies, whereas global alignments, e.g. LAGAN (Brudno et al. 2003), AVID (Bray et al. 2003), enable pairwise global comparisons of very large genomic regions (at megabase scale sequences). Once the orthologous non-coding sequence is located near the gene, then the best conserved motifs in those homologous regions are identified independently (see Blanchette and Tompa, 2003). The second group of methods does not assume that the orthologous sequences can be reliably aligned, but instead directly attempts to identify motifs that exhibit a high degree of conservation. More recent algorithms integrate these two approaches, making use of local multiple sequence alignment blocks when these are available and reliable, but also allowing the detection of motifs in unalignable regions (Fang and Blanchette, 2006).

In both alignment-based and motif-finding approaches, the central assumption is that functional sequences evolve under constraints while non-functional sequences evolve neutrally. For this part of the analysis, an appropriate evolutionary model will be of crucial importance. However the underlying evolutionary models used in some of the methods described above suffer from one or more limitations. Some methods can only be applied to two species, some treat orthologous sequences as statistically independent, and some neglect the divergence time among species (not really exploiting all the historical information). Several algorithms have been developed recently that take into account the phylogenetic relationships of the species under consideration. For example, EMnEM (Moses et al. 2003) uses a Jukes Cantor model in which the substitution rate inside the regulatory element is fixed, ignoring the positional variation of the motif. PhyME (Sinha et al. 2004) and PhyloGibbs (Siddharthan et al. 2005) use a model similar to Felsenstein’s molecular evolution model (Felsenstein, 1981), which combines binding site specificity with substitution rate. More detailed models have also been described (e.g. Li et al. 2005; Gertz et al. 2006) that improve the accuracy and confidence of computational predictions of functional motifs.

### Construction/comparison of networks/pathways

**Figure 2:**
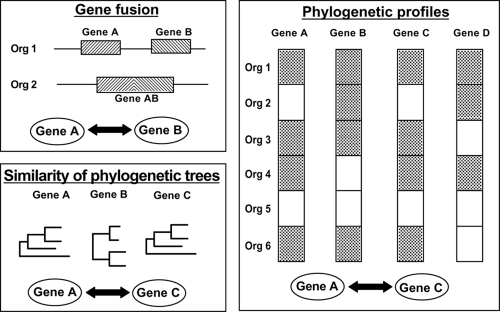
Methods used for the prediction of protein-protein interactions.

In the post-genomic view of cellular function, each biological entity is seen in the context of a complex network of interactions. New and powerful experimental techniques, such as the yeast two-hybrid system or tandem-affinity purification and mass spectrometry, are used to determine protein-protein interactions systematically. Nevertheless, information on protein—protein interactions is still mostly limited to a small number of model organisms. Furthermore, it has recently been estimated that the overall average false positive rate of available computational and high-throughput experimental interaction datasets is as high as 90%. Therefore, a number of computational techniques have been designed for predicting and scoring protein interactions on the genome scale (see Fig. 2).

Proteins that interact are assumed to be more likely to co-evolve, therefore it is possible to make inferences about interactions between pairs of proteins based on phylogenetic relationships. For example, the Rosetta method relies on the observation that some interacting proteins have homologs in another organism fused into a single protein chain (Marcotte et al. 2001). Pellegrini et al. 1999 introduced another method based on the property of correlated evolution, by characterizing each protein by its phylogenetic profile, a string that encodes the presence or absence of a protein in every known genome. A measure of the similarity between phylogenetic trees of protein families has also been used to predict pairs of interacting proteins (Pazos and Valencia, 2001). This method was adapted to consider the multi-domain nature of proteins by breaking the sequence into a set of segments of predetermined size and constructing a separate profile for each segment (Kim and Subramaniam, 2006).

Methods have also been developed to predict the interaction surface or specific residues. One approach involves quantifying the degree of covariation between residues from pairs of interacting proteins (correlated mutations), known as the “in silico two-hybrid” method. For certain proteins that are known to interact, correlated mutations have been demonstrated to be able to select the correct structural arrangement of two proteins based on the accumulation of signals in the proximity of interacting surfaces (Pazos et al. 1997). This relationship between correlated residues and interacting surfaces has been extended to the prediction of interacting protein pairs based on the differential accumulation of correlated mutations between the interacting partners (interprotein correlated mutations) and within the individual proteins (intra-protein correlated mutations) (Pazos and Valencia, 2002).

### Phylogenetic analyses at the genome level: genome evolutionary mechanisms

In the new era of genomics, fresh perspectives are opening to scientists seeking to unravel the evolutionary mechanisms that shape contemporary genomes and to reconstruct ancestral genomes. Reconstruction can be approached at different levels depending on the time scale and the available genomic data (reviewed in Rascol et al. 2007). For example, a number of authors (Murphy et al. 2005; Bourque et al. 2005; Ma et al. 2006) have studied mammalian chromosomal evolution and have described the architecture of the ancestral mammalian genome. Several attempts have also been conducted to perform reconstruction deeper in the tree of life, at the vertebrate level (Jaillon et al. 2004; Kohn et al. 2006). Reconstruction of ancestral prokaryotic genomes has indicated the dominance of horizontal gene transfer in the evolution of prokaryotes (Mirkin et al. 2003; Dagan and Martin, 2007). Reconstructions of more distant species are more difficult due to numerous genomic events, such as chromosomal rearrangements accumulating during the history of species. In future studies, the reconstruction process should be greatly enhanced with the availability of additional phylogenetically informative genomes, and the possibility of exploring important intermediate nodes.

The detection of local conservation of gene content and proximity across several genomes are of crucial importance not only toward a full understanding of the forces that shaped our genome, but also in predicting important features of interest, such as the physical interaction of proteins or their participation in common metabolic/regulatory networks (e.g. Marcotte et al. 1999; Enright and Ouzounis, 2001; von Mering et al. 2003). For instance, long-range enhancers and their regulatory target genes have been found in chromosomal segments, representing loci that maintain syntenic relationships through all vertebrate genomes. The target genes within these segments as well as their inferred *cis*-regulatory sequences are likely to be fundamental to general vertebrate development and ontogeny (Kikuta et al. 2007). A notable example is the coregulated *hox* clusters that are conserved throughout most metazoan genomes (Lee et al. 2006). A growing number of pathologies have also been directly or indirectly linked to features of genome architecture. Genomic rearrangements may cause Mendelian diseases, produce complex traits such as behaviors, or represent benign polymorphic changes. The mechanisms by which rearrangements convey phenotypes are diverse and include gene dosage, gene interruption, generation of a fusion gene, position effects and unmasking of recessive coding region mutations (single nucleotide polymorphisms, SNPs, in coding DNA) or other functional SNPs (Lupski and Stankiewicz, 2005). For example, recent findings suggest that segmental duplications, a common architectural feature of many genomes, have had important roles in creating novel primate gene families, and in shaping the human genetic variation that is thought to contribute significantly to disease susceptibility (Bailey and Eichler, 2006).

## Perspectives

One of the main objectives over the last decade has been the study of the mechanisms involved in the evolution of the genome and their consequences in the study of biological systems. In order to gain a clearer understanding of the fundamental aspects of the evolutionary process and the factors that shape contemporary genes and genomes, efficient treatment of the vast amounts of genomic data will be required, based on a pertinent use of the phylogenetic approaches we have described in this review.

Unfortunately, the vast number and complexity of the events shaping eukaryotic genomes means that a complete understanding of evolution at the genomic level is not currently feasible. At the lowest level, point mutations affect individual nucleotides. At a higher level, large chromosomal segments undergo duplication, lateral transfer, inversion, transposition, deletion and insertion. Ultimately, whole genomes are involved in processes of hybridization, polyploidization and endosymbiosis, often leading to rapid speciation. Various approaches, known as phylogenomic approaches, have been used to reconstruct a tree of life by using the maximum of available genomic data (reviewed in Wolf et al. 2002). These include methods based on gene content, gene order, evolutionary distances between orthologs, concatenated alignments of orthologous protein sequences and a combination of multiple, independently reconstructed trees. The different topologies resulting from these studies suggest that the notion of a single Tree of Life that would accurately depict the evolution of all life forms is over simplistic. Individual genes possess their own, unique evolutionary histories, due to the fact that different genes have evolved at different points during the history of life and that, in addition to vertical inheritance, evolution of many orthologous families involved lineage-specific gene loss and gene acquisition by horizontal transfer. As a consequence, it has been suggested that, at least for prokaryotes, the phylogenetic history of genomes should be represented as a bush or a network, rather than a tree (Wolf et al. 2002).

**Figure 3:**
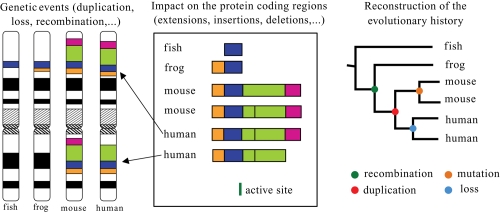
Reconstruction of evolutionary histories.

An exhaustive comparison of the whole sets of proteins (proteomes) encoded by completely sequenced genomes will be crucial to understanding genome evolution by taking into account the major mechanisms occurring at the gene level. The modular structure of the contemporary protein will then allow us to trace back the successive events of ancestral gene duplication and fusion of evolutionarily unrelated genes which occurred at different periods and thus, to reconstruct the ancestral genes which were at the origin of the family (see Fig. 3). Some work has already been performed in this area, both for prokaryotic genomes (e.g. Mirkin et al. 2003; Descorps-Declère et al. 2007) and for eukaryotic genomes (e.g. Koonin et al. 2004).

The methodologies now being developed in this context should facilitate the efficient exploitation of evolutionary information in functional genomics (notably, in interactomics and transcriptomics comparisons or in high throughput promoter studies) and large scale systems biology projects. Currently, the use of evolutionary concepts is underexploited and in future such expertise could be integrated for phylogenetic reconstruction and functional inference. In particular conserved regions could be highly informative for phylogenetic inference. In the longer term, such methodologies should also facilitate the automated integration and analysis of evolutionary features introduced by newly sequenced genomes. Such advances will be fundamental for the development of new fields such as systems biology and synthetic biology. This is equally important to progress in applied fields such as biotechnology, agronomy, medicine and pharmacology.
